# High myopia in Greater Beijing School Children in 2016

**DOI:** 10.1371/journal.pone.0187396

**Published:** 2017-11-09

**Authors:** Yin Guo, Jia Li Duan, Li Juan Liu, Ying Sun, Ping Tang, Yan Yun Lv, Liang Xu, Jost B. Jonas

**Affiliations:** 1 Tongren Eye Care Center, Beijing Tongren Hospital, Capital Medical University, Beijing, China; 2 Beijing Institute of Ophthalmology and Visual Science Key Lab, Beijing Tongren Eye Center, Beijing Tongren Hospital, Capital Medical University, Beijing, China; 3 Beijing Center for Disease Prevention and Control, Beijing, China; 4 Department of Ophthalmology, Medical Faculty Mannheim of the Ruprecht-Karls-University Heidelberg, Seegartenklinik Heidelberg, Germany; LV Prasad Eye Institute, INDIA

## Abstract

**Purpose:**

To assess prevalence and associated factors of myopia and high myopia in schoolchildren in Greater Beijing.

**Methods:**

The school-based, cross-sectional Greater Beijing School Children Myopia study was carried out in the year 2016 in 54 schools randomly selected from 15 districts in Beijing. Non-cycloplegic auto-refractometry of the right eyes was performed.

**Results:**

The study included 35,745 (99.4%) out of 35,968 eligible pupils with a mean age of 12.6±3.4 years (range 6–18 years). Prevalence of myopia defined as myopic refractive error of ≥-0.50 diopters (D),≥-1D,≥-6D,≥-8D and ≥-10D was 70.9%(95% confidence intervals (CI):70.5,71.4), 60.9% (95%CI:60.4,61.4), 8.6%(95%CI:8.4,8.9), 2.2%(95%CI:2.0,2.4), and 0.3% (95%CI:0.3,0.4), respectively. The frequency of high myopia (≥-6D, ≥-8D, ≥-10D) increased from 1.5% (95%CI:1.0,2.0), 0.4% (95%CI:0.1,0.6) and 0.1% (95%CI:0.00,0.02), respectively in 10-year-olds to 19.4% (95%CI:17.3,21.6), 5.2% (95%CI:4.0,6.4) and 0.9% (95%CI:0.4,1.5), respectively, in 18-year-olds. Mean refractive error in the 18-year-olds was -3.74±2.56D (median:-3.63D;range:-19.6D to + 6.25D). Higher prevalence of high myopia (≥-6D and ≥-8D) was correlated (all *P*<0.001) with older age (OR:1.18, and 1.15, respectively), female gender (OR: 1.44 and 1.40, respectively), higher body mass index (OR: 1.02 and 1.03, respectively), taller body height (OR: 1.03 and 1.02, respectively), urban region of habitation (OR: 1.26 and 1.33, respectively) and higher school type (OR:1.57 and 2.22, respectively). Prevalence of severe high myopia (≥-10D) was associated only with older age (*P*<0.001; OR: 1.44; 95%CI: 1.31, 1.59) but not with any education-related parameter such as higher school type (*P* = 0.48), urban region of habitation (*P* = 0.07) or female gender (*P* = 0.37).

**Conclusion:**

In this most recent survey, prevalence of high myopia (≥-6D:19.4%;≥-8D:5.2%;≥-10D:0.9%) in 18-year-old school children was higher than in previous surveys from mainland China. In contrast to minor high myopia and moderate high myopia (defined as myopic refractive error of <-10D), severe high myopia (myopic refractive error ≥-10D) was not strongly correlated with educational parameters.

## Introduction

Recent investigations have convincingly shown the marked increase in the prevalence of myopia among the young generation worldwide and especially in East Asia [1–14]. Since high myopia can lead myopic maculopathy and high myopia-associated glaucomatous optic neuropathy, the risk has risen that myopia may become the main blinding disease worldwide in the future [15–17]. This notion has been based on the findings that myopic maculopathy was one of the most common causes for visual impairment and blindness in the elderly population of the Beijing Eye Study already in 2001, that the marked increase in the prevalence of myopia has just arrived in the young and middle-aged generations in China, and that it will need one to two decades more to reach the age groups in which myopia-related complications including myopic maculopathy and optic nerve damage usually become clinically manifest [18]. It is therefore important to develop preventive measures against myopia, and in particular against high myopia. Since preceding studies on the prevalence of myopia in the school children generation of China dated back several years, since they included usually less than 5,000 children, and in particular since these studies did not specifically focus on high myopia, which is the myopia group with the highest risk of eventual myopia-related complications, we carried out the present study to assess the prevalence of high myopia in a relatively large group of school children attending randomly selected schools in Greater Beijing.

## Methods

The Greater Beijing School Children Myopia Study was a school-based, cross-sectional investigation conducted in 2016 in Greater Beijing, China. It was approved by the ethics committee of the Capital Medical University, Beijing Center for Disease Control and Prevention and followed the Declaration of Helsinki. After explanation of the study design to parents and children, informed written consent was obtained from at least one parent per child. The study was carried out in 54 schools which were differentiated into the primary level, junior level and senior level and which were randomly selected from 5 urban districts of Beijing (Dongcheng, Xicheng, Chaoyang, Haidian, Fengtai) and from 10 rural districts of Greater Beijing (Tongzhou, Changping, Mentougou, Fangshan, Huairou, Pinggu, Shunyi, Miyun, Yanqing, Shijingshan). Due to selecting the schools randomly, performed separately in the rural region and in the urban region, the study population was a representative sample of the school children population in Greater Beijing. In each school, all children aged 6 to 18 years participated in the study and underwent measurement of uncorrected visual acuity and auto refractometry (Topcon RM-A7000; Topcon Co, Tokyo, Japan) without cycloplegia. Refractometry was performed thrice, and the average of all three measurements was taken for further statistical analysis. Additionally, we determined body height and body weight and calculated the body mass index (BMI) as the ratio of body weight (kg) divided by the square of body height (m). Using a stadiometer, body height was measured with the shoes removed. The children were asked to stand upright as much as possible and with the head raised upright as much as possible. The examinations were carried out by four to five junior doctors who had specifically been trained in the examination techniques before start of the study. Since the examiners worked together mostly in the same rooms, the surveyed each other and were additionally supervised by principal investigators (YG, LX).

Myopia was defined as myopic refractive error (spherical equivalent) of ≥-0.50 diopters and ≥-1.00 diopters, respectively. The reason to use also the second definition of myopia with a myopic refractive error of more than -1.00 diopters was that refractometry was carried out without cycloplegia so that due to involuntary accommodation the refractive measurements might have been artificially low in some children. Minor high myopia was defined as a refractive error of ≥-6.00 diopters, moderate high myopia was defined as a myopic refractive error ≥-8.00 diopters, and severe high myopia was defined as a myopic refractive error ≥-10.00 diopters, respectively.

The statistical analysis was performed using a commercially available software package (SPSS for Windows, version 22.0, IBM-SPSS, Chicago, IL). We calculated the mean ± standard deviation of refractive error (presented as spherical equivalent) and presented the prevalence of myopia as mean and the 95% confidence intervals (CI). Logistic regression was used to assess the relationship between prevalence of myopia and parameters such as age, gender, body height, BMI, school type and school grade. With the prevalence of myopia as dependent variable, we first conducted a univariate analysis, followed by a multivariate analysis, which included all parameters as independent variables which were significantly correlated with the prevalence of myopia in the univariate analysis. We calculated odds ratios (OR) and their 95% confidence intervals (CI). All *P*-values were two-sided and considered statistically significant if <0.05.

## Results

The study included 35,745 (99.4%) out of 35,968 eligible school children (18,276 (51.1%) boys), with 8,090 children (22.6%) living in the urban regions. The mean age was 12.6±3.4 years (range: 6–18 years). Primary school was attended by 13,241 students (37.0%), the junior school by 11,254 (31.5%) students, and the senior school by 11,283 (31.5%) students. The mean body height and body weight were 156.0±16.7cm and 52.0±16.9kg, respectively. (Table 1)

**Table 1:** Demographic characteristics (mean ± standard deviation) of the participants in the Greater Beijing School Children Myopia Study 2016.

Age (Years)	n (%)	Refractive Error (Diopters)	Body Height (cm)	Body Weight (kg)	Body Mass Index (kg(m^2^)	Uncorrected Visual Acuity (logMAR) Right Eye	Uncorrected Visual Acuity (logMAR) Left Eye
6	1231 (3.4)	0.20 ± 0.86	121.9 ± 5.2	24.5 ± 5.6	16.4 ± 2.9	0.03 ± 0.11	0.04 ± 0.12
7	1911 (5.3)	-0.03 ± 0.98	126.9 ± 6.0	27.1 ± 6.6	16.6 ± 3.1	0.03 ± 0.15	0.03 ± 0.14
8	2285 (6.4)	-0.31 ± 1.12	132.7 ± 5.8	31.1 ± 8.0	17.5 ± 3.5	0.06 ± 0.19	0.05 ± 0.18
9	1874 (5.2)	-0.71 ± 2.09	138.4 ± 6.7	35.1 ± 9.7	18.1 ± 3.7	0.12 ± 0.27	0.11 ± 0.26
10	1848 (5.2)	-1.14 ± 1.76	144.8 ± 7.1	40.6 ± 10.9	19.1 ± 4.0	0.19 ± 0.31	0.18 ± 0.31
11	2127 (5.9)	-1.60 ± 1.89	150.7 ± 7.4	45.8 ± 12.5	19.9 ±4.3	0.26 ± 0.34	0.24 ± 0.33
12	2414 (6.7)	-2.14 ± 2.11	157.8 ± 7.5	52.3 ± 13.6	20.8 ± 4.5	0.33 ± 0.36	0.31 ± 0.35
13	4221 (11.8)	-2.44 ± 2.21	162.3 ± 7.5	56.4 ± 14.4	21.2 ± 4.5	0.38 ± 0.37	0.35 ± 0.37
14	3433 (9.6)	-2.87 ± 2.28	165.7 ± 7.7	60.4 ± 14.9	21.9 ± 4.6	0.47 ± 0.37	0.43 ± 0.38
15	3972 (11.1)	-3.29 ± 2.42	167.5 ± 8.0	62.8 ± 15.0	22.3 ± 4.5	0.51 ± 0.36	0.47 ± 0.37
16	3967 (11.1)	-3.65 ± 2.53	168.3 ± 8.4	64.2 ± 15.3	22.6 ± 4.5	0.56 ± 0.35	0.51 ± 0.37
17	3226 (9.0)	-3.89 ± 2.59	168.4 ± 8.5	64.5 ± 15.5	22.6 ± 4.5	0.59 ± 0.35	0.55 ± 0.37
18	1264 (3.5)	-3.74 ± 2.56	168.5 ± 8.7	64.5 ± 15.4	22.6 ± 4.4	0.57 ± 0.36	0.54 ± 0.38

**Fig 1:**
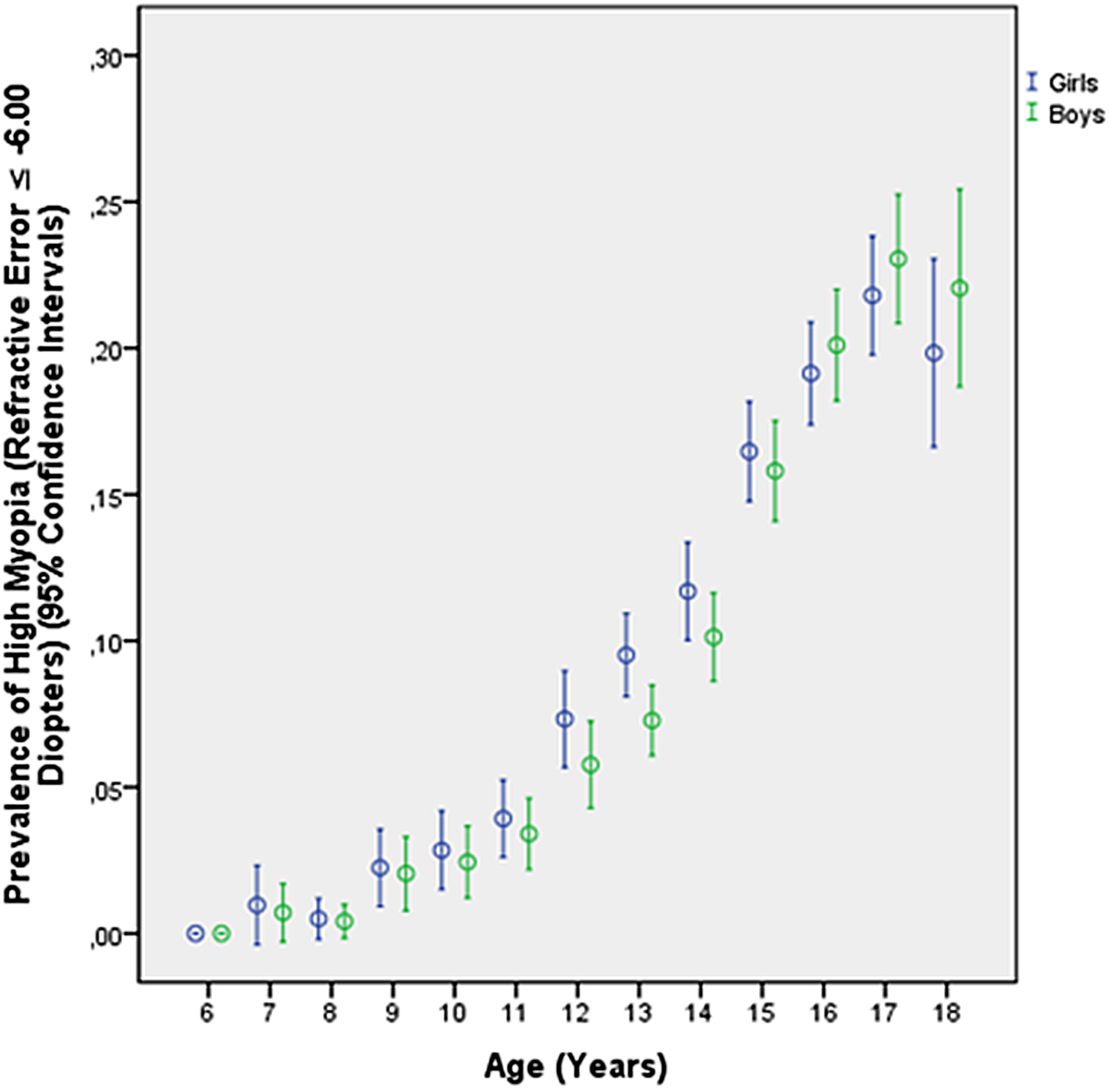
Diagram showing the prevalence of minor high myopia (myopic refractive error ≥-6.00 diopters) in the Greater Beijing School Children Myopia Study 2016.

**Fig 2:**
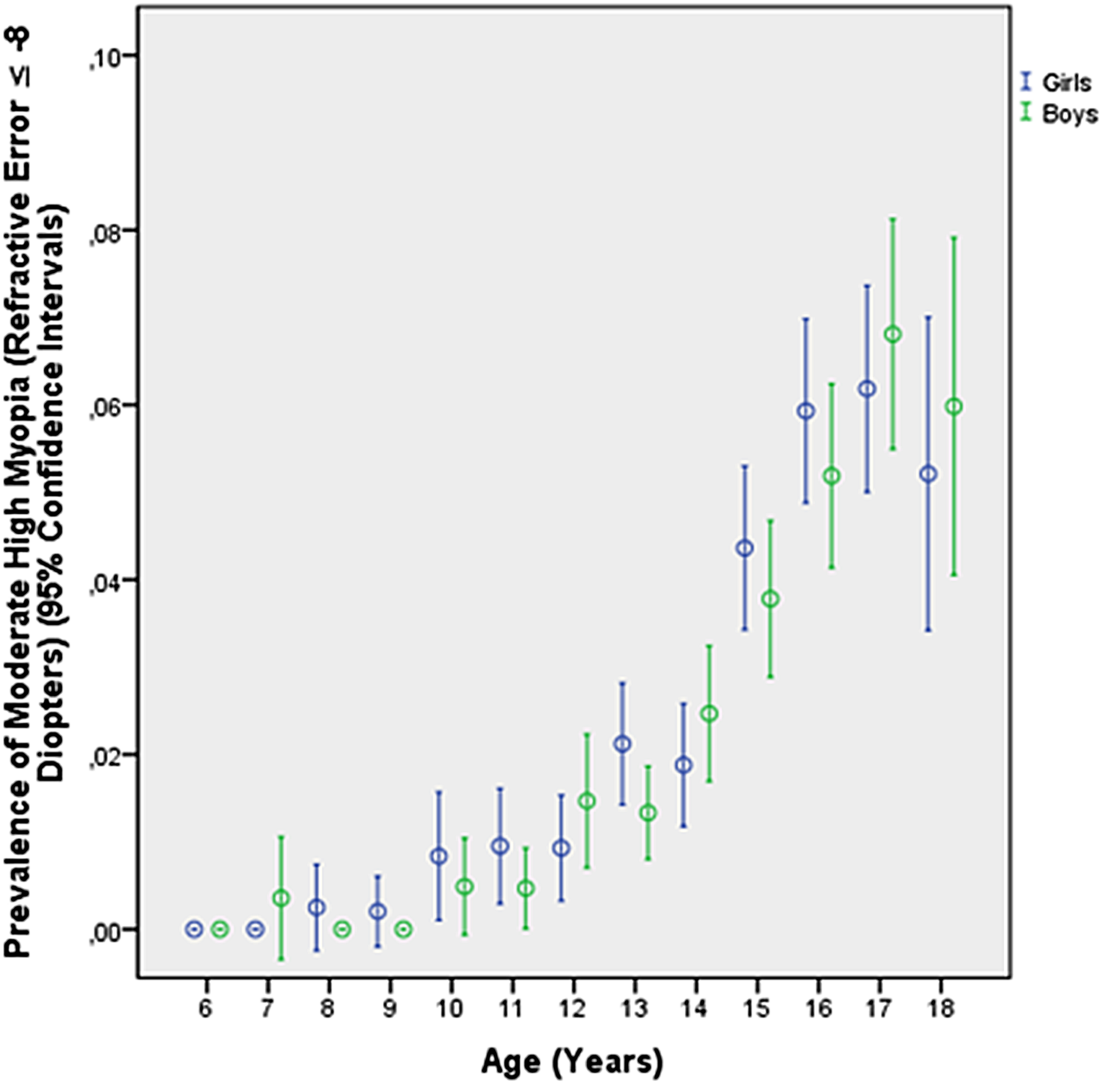
Diagram showing the prevalence of moderate high myopia (myopic refractive error ≥-8.00 diopters) in the Greater Beijing School Children Myopia Study 2016.

**Fig 3:**
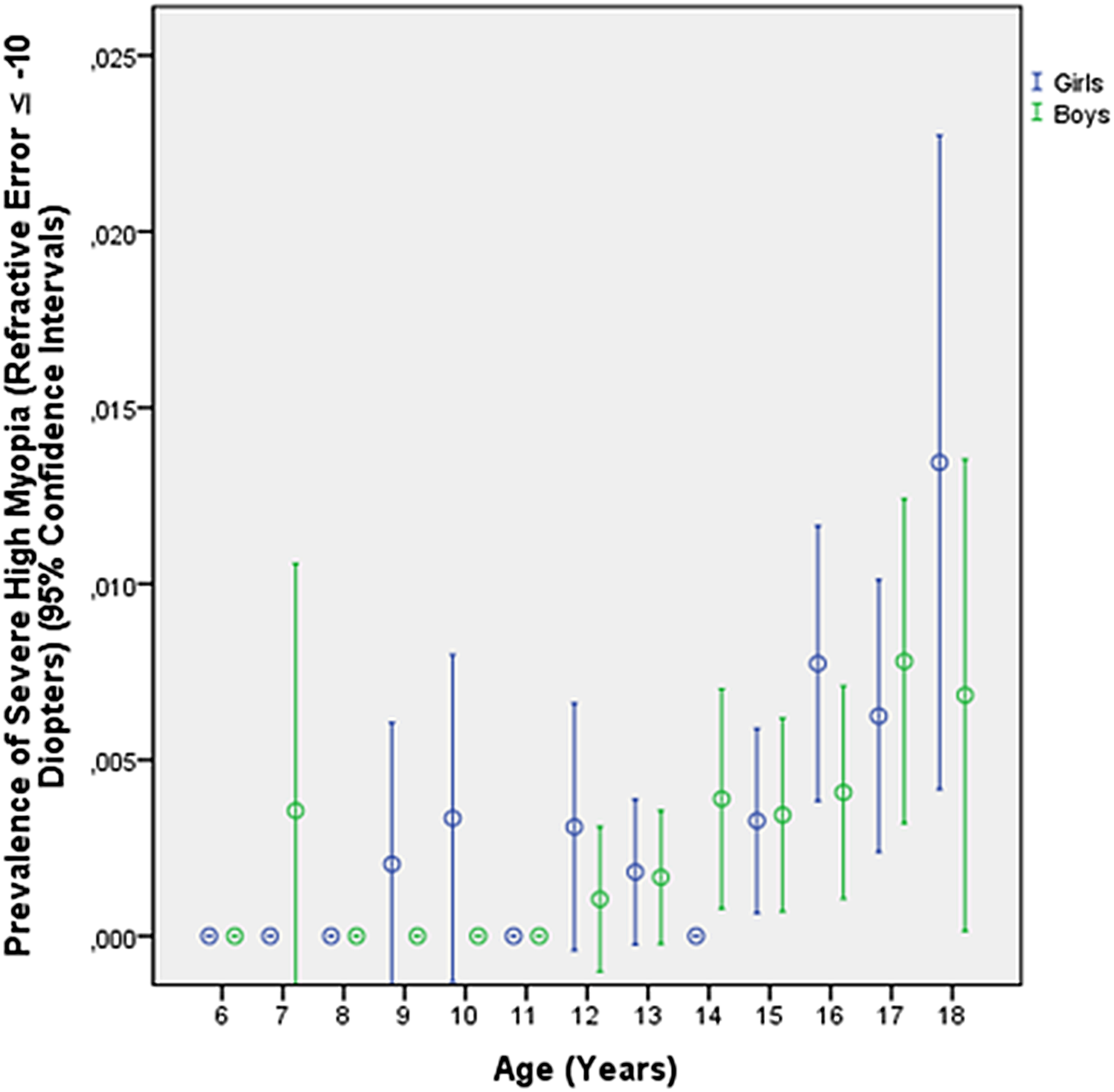
Diagram showing the prevalence of severe high myopia (myopic refractive error ≥-10.00 diopters) in the Greater Beijing School Children Myopia Study 2016.

**Fig 4:**
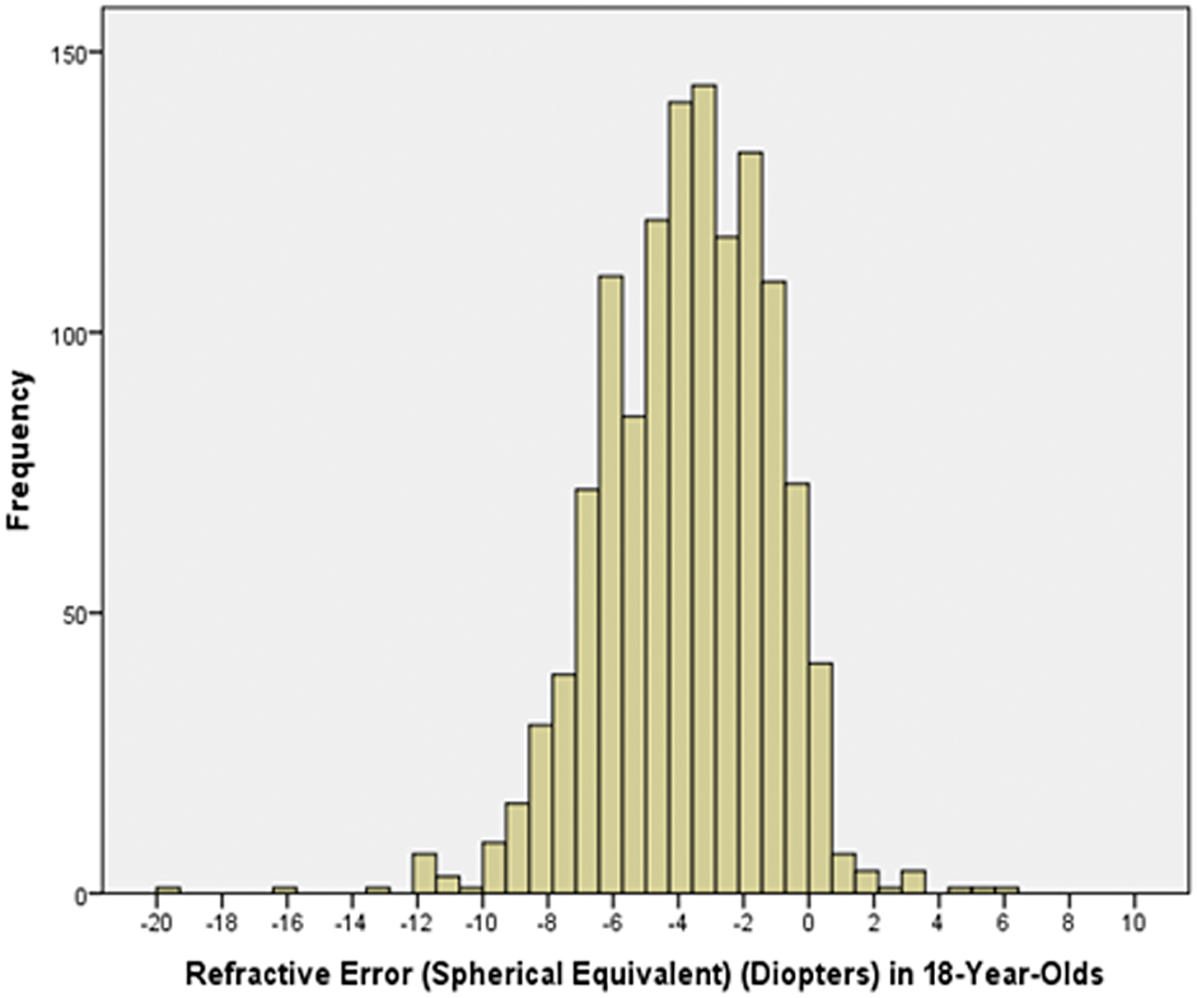
Histogram showing the distribution of refractive error in the 18year-olds in the Greater Beijing School Children Myopia Study 2016.

Prevalence of myopia defined as myopic refractive error (spherical equivalent) of ≥-0.50 diopters, ≥-1.00 diopters, ≥-6.00 diopters (Table 2), ≥-8.00 diopters and ≥-10.00 diopters was 70.9% (95% confidence intervals (CI): 70.5, 71.4), 60.9% (95%CI: 60.4, 61.4), 8.6% (95%CI: 8.4, 8.9), 2.2% (95%CI: 2.0, 2.4), and 0.3% (95%CI: 0.3, 0.4), respectively. The frequency of minor high myopia (≥-6.0 diopters) increased from 1.51% (95%CI: 1.0, 2.0) in 10-year-olds to 19.4% (95%CI: 17.3, 21.6) in 18-year-old teenagers (Table 2; Fig 1), the prevalence of moderate high myopia (≥-8.00 diopters) increased from 0.4% (95%CI: 0.1, 0.6) in 10-year-olds to 5.2% (95%CI: 4.0, 6.4) in 18-year-olds (Fig 2), and the prevalence of severe high myopia (≥-10.00 diopters) increased from 0.1% (95%CI: 0.00, 0.02) in 10-year-olds to 0.9% (95%CI: 0.4, 1.5) in 18-year-olds (Fig 3). Mean refractive error in the 18-year-olds was -3.74 ± 2.56 diopters (median: -3.63 diopters; range: -19.6 to + 6.25 diopters) (Fig 4).

**Table 2:** Prevalence of myopia defined as refractive error ≥-6.00 diopters stratified by age, gender and region of habitation in the Greater Beijing School Children Myopia Study 2016.

Age (Years)	Total	Boys	Girls	Urban Region	Rural Region
6	0/1415 (0%)	0/734 (0%)	0/681 (0%)	0/47 (0%)	0/1368 (0%)
7	4/2168 (0.2%)	2/1079 (0.2%)	2/1089 (0.2%)	0/180 (0%)	4/1988 (0.2%)
8	4/2592 (0.2%)	2/1357 (0.1%)	2/1235 (0.2%)	1/213 (0.5%)	3/2379 (0.1%)
9	21/2163 (0.9%)	10/1096 (0.9%)	11/1067 (1.0%)	4/166 (2.4%)	17/1997 (0.9%)
10	32/2118 (1.5%)	15/1142 (1.3%)	17/976 (2.7%)	5/194 (2.6%)	27/1924 (1.4%)
11	62/2476 (2.5%)	29/1322 (2.2%)	33/1154 (2.9%)	9/215 (4.2%)	53/2261 (2.3%)
12	126/2497 (5.0%)	55/1296 (4.2%)	71/1201 (5.9%)	37/708 (5.2%)	89/1789 (5.0%)
13	288/4248 (6.8%)	131/2275 (5.8%)	157/1973 (7.9%)	110/1330 (8.3%)	178/2918 (6.1%)
14	324/3458 (9.4%)	156/1845 (8.5%)	168/1613 (10.4%)	141/1082 (13.0%)	183/2376 (7.7%)
15	578/3993 (14.5%)	276/1978 (13.9%)	302/2015 (14.9%)	210/1254 (16.7%)	368/2739 (13.4%)
16	716/3977 (18.0%)	345/1910 (18.1%)	371/2067 (17.9%)	235/1229 (19.1%)	481/2748 (17.5%)
17	674/3242 (20.8%)	325/1545 (21.0%)	349/1697 (20.6%)	217/1020 (21.2%)	457/2222 (20.6%)
18	247/1271 (19.4%)	129/632 (20.4%)	118/639 (18.5%)	97/421 (23.0%)	150/850 (17.6%)

In univariate analysis, higher prevalence of minor high myopia (≥-6 diopters) was associated with older age (*P*<0.001), urban region of habitation (*P*<0.001), higher BMI (*P*<0.001), higher body height (*P*<0.001), female gender (*P*<0.001), higher school type (*P*<0.001), and higher school grade (*P*<0.001). In the multivariate binary regression analysis with prevalence of minor high myopia as dependent variable, we dropped school grade due to collinearity with age. In the final model, higher prevalence of minor high myopia was correlated (all *P*<0.001) with older age, female gender, higher BMI, taller body height, urban region of habitation and higher school type (Table 3). Similar results were obtained for the prevalence of moderate high myopia defined as refractive error ≥-8 diopters (Table 3). Higher prevalence of high myopia (if defined as ≥-9 diopters) was correlated with older age (*P* = 0.006), higher BMI (*P* = 0.01), urban region of habitation (*P* = 0.004) and higher school type (*P*<0.001), while body height (*P* = 0.13) and gender (*P* = 0.09) were no longer significantly associated (Table 3). If children with a myopic refractive error of ≥-10 diopters were excluded, similar results were obtained, with a higher prevalence of high myopia (here defined as ≥-8 diopters and ≤-9 diopters) being associated with older age (*P*<0.001; OR: 1.18; 95%CI: 1.14, 1.23), female gender (*P*<0.001; OR: 1.45; 95%CI: 1.31, 1.60), higher BMI (*P*<0.001; OR: 1.02; 95%CI:1.01, 1.03), taller body height (*P*<0.001; OR: 1.03; 95%CI: 1.02, 1.03), urban region of habitation (*P*<0.001; OR: 1.24; 95%CI: 1.14, 1.35) and higher school type (*P*<0.001; OR: 1.48; 95%CI: 1.29, 1.71).

**Table 3:** Associations (multivariate analysis) of the prevalence of high myopia with different definition (≥-6 diopters, ≥-8 diopters, ≥-9 diopters,) in the Greater Beijing School Children Myopia Study 2016, in the whole study population and stratified by gender.

	≥-6 diopters (n = 3090)				≥-8 diopters (n = 784)				≥-9 diopters (n = 361)			
	*P*-Value	Odds Ratio	95% CI		*P*-Value	Odds Ratio	95% CI		*P*-Value	Odds Ratio	95% CI	
			Lower	Upper			Lower	Upper			Lower	Upper
Age (Years)	<0.001	1.18	1.14	1.23	<0.001	1.15	1.06	1.23	0.006	1.15	1.04	1.28
Gender (Girls / Boys)	<0.001	1.44	1.31	1.58	<0.001	1.40	1.16	1.68				
Body Mass Index (kg/m^2^)	<0.001	1.02	1.01	1.03	0.001	1.03	1.01	1.04	0.01	1.03	1.01	1.05
Body Height (cm)	<0.001	1.03	1.02	1.03	<0.001	1.02	1.01	1.03				
Urban (= 1), Rural (= 2) Region of Habitation	<0.001	1.26	1.16	1.37	<0.001	1.33	1.14	1.54	0.004	01.38	1.11	1.71
School Type	<0.001	1.57	1.37	1.79	<0.001	2.22	1.71	2.89	<0.001	2.68	1.82	3.94
Boys												
	≥-6 diopters (n = 1485)				≥-8 diopters (n = 372)				≥-9 diopters (n = 165)			
	*P*-Value	Odds Ratio	95% CI		*P*-Value	Odds Ratio	95% CI		*P*-Value	Odds Ratio	95% CI	
			Lower	Upper			Lower	Upper			Lower	Upper
Age (Years)	<0.001	1.21	1.14	1.28	<0.001	1.22	1.10	1.36	0.001	1.29	1.11	1.50
Body Mass Index (kg/m^2^)	0.003	1.02	1.01	1.03	0.008	1.03	1.01	1.05	0.02	1.04	1.01	1.07
Body Height (cm)	<0.001	1.03	1.02	1.03	0.04	1.02	1.01	1.03				
Urban (= 1), Rural (= 2) Region of Habitation	<0.001	1.29	1.14	1.45	0.03	1.27	1.02	1.57				
School Type	<0.001	1.50	1.24	1.82	0.002	1.82	1.25	2.66	0.04	1.78	1.02	3.09
Girls												
	≥-6 diopters (n = 1605)				≥-8 diopters (n = 412)				≥-9 diopters (n = 196)			
	*P*-Value	Odds Ratio	95% CI		*P*-Value	Odds Ratio	95% CI		*P*-Value	Odds Ratio	95% CI	
			Lower	Upper			Lower	Upper			Lower	Upper
Age (Years)	<0.001	1.16	1.10	1.23	0.03	1.11	1.01	1.23	0.001	1.43	1.34	1.53
Body Mass Index (kg/m^2^)	0.003	1.02	1.01	1.03	0.03	1.03	1.00	1.05				
Body Height (cm)	<0.001	1.03	1.02	1.04								
Urban (= 1), Rural (= 2) Region of Habitation	<0.001	1.25	1.11	1.40	0.001	1.42	1.15	1.76	0.001	1.66	1.24	2.22
School Type	<0.001	1.63	1.35	1.97	<0.001	2.89	1.99	4.20				

In univariate analysis, higher prevalence of severe high myopia (≥-10 diopters) was associated with older age (*P*<0.001; OR: 1.45; 95%CI: 1.32, 1.61), higher BMI (*P*<0.001; OR: 1.09; 95%CI: 1.05, 1.13), taller body height (*P*<0.001; OR: 1.05; 95%CI: 1.02, 1.07), school grade (*P*<0.001; OR: 1.46; 95%CI: 1.32, 1.62), school type (*P*<0.001; OR: 3.63; 95%CI: 2.54, 5.17) and with urban region of habitation (*P*<0.001; OR: 2.20; 95%CI: 1.43, 3.38), but not with gender (*P* = 0.24; OR: 1.29; 95%CI: 0.84, 1.97). Since all these parameters were significantly and strongly (all *P*<0.001) correlated with age, the associations were adjusted for age. After adjusting for age, prevalence of severe high myopia was no longer significantly with BMI (*P* = 0.12; OR: 1.03; 95%CI: 0.99, 1.08), body height (*P* = 0.62; OR: 1.01; 95%CI: 0.98, 1.03), region of habitation (*P* = 0.07; OR: 1.49; 95%CI: 0.97, 2.30) or school type (*P* = 0.48; OR: 1.31; 95%CO: 0.62, 2.76), and gender (*P* = 0.37; OR: 1.21; 95%CI: 0.80, 1.86).

## Discussion

In this large-scaled population-based study on school children in urban and rural Greater Beijing, the prevalence of high myopia (≥-6 diopters,≥-8 diopters, ≥-10 diopters) in 18-year-olds was 19.4% (95%CI:17.3,21.6),5.2% (95%CI:4.0,6.4) and 0.9% (95%CI:0.4,1.5), respectively. While the prevalence of high myopia defined as refractive error of ≥-6 diopters up to <-10 diopters was associated with education-related parameters (such as higher school type, urban region of habitation, and female gender) after adjusting for age, the frequency of severe high myopia defined as myopic refractive error of ≥-10 diopters was not correlated with educational parameters after adjusting for age.

The prevalence of minor and moderate high myopia in our study was higher than in most previous studies on Chinese school children. It included the study by conducted Fan and colleagues in Hong Kong, the Shunyi study in a rural region of Greater Beijing, an investigation performed in Guangzhou / South China, a nation-wide survey in Taiwan, the Xichang Pediatric Refractive Error Study conducted in Guangdong province in South China, the Beijing Childhood Eye Study, the Shandong Children Eye Study and the Gobi Desert Children Eye Study, to name only a few (Table 4). The results from these studies conducted in China were complemented by findings of investigations carried out in other East Asian countries (Table 4). The general tendency towards a higher prevalence of myopia in the younger generation in China has also been demonstrated in a recent investigation by Xiang and colleagues who showed that the prevalence of myopia was significantly higher in Chinese children than in their parents [13]. A similar result was reported in the Handan Offspring Myopia Study [14].

**Table 4:** Summary of studies related to myopia/high myopia.

	Place	Year of Study	Location (Rural/Urban)	Number of Participants	Age (Years)	Definition of Myopia	Prevalence
Fan et al. [1]	Hong Kong	1998–2000		7560	9.33	≥-6.0D	1.19%
Zhao et al. [2]	Shunyi District, Beijing	1999	rural	5884	5 and 15	≥-0.50D:	5years:0% 15 years Boys:36.7% Girls:55.0%
He et al. [3,5]	Guangzhou	2002/2003	Rural/Urban	4364	15	≥-6.0D	4.8%
Lin et al. [4]	Taiwan	1983 to 2000		45345	18	≥-6.0D	1983: 10.9% 2000: 21%
Congdon et al. [6]	Guangzhou	2007	rural	1892	14.7	>−6.0	1.9%
Sun et al. [7]	Shanghai	2009		5083	20.2	≥-6 D	19.5%
You et al. [8]	Beijing	2011	Rural/Urban	15066	13.2	≥-6 D ≥-8 D	4.3% 1.0%
Wu et al. [9]	Shandong	2012/2013		6026	9.7	≥-6 D	2.0%
Guo et al. [10]	Ejina	2013		1565	11.9	≥-6 D	2.9%
Wu et al. [11]	Beijing	2015		4677	16.9	≥-6 D	9.7%
Jung et al. [12]	Seoul	2010		23616	19	≥−0.5D	96.5%

Compared with all previous studies from mainland China, the present investigation suggested a further increase in the prevalence of minor high myopia defined as a myopic refractive error of ≥-6 diopters. Since the present study was the first investigation reporting on the prevalence of moderate high myopia defined as myopic refractive error of ≥-8 diopters and of severe high myopia (≥-10 diopters), conclusions on a change in the prevalence of moderate high myopia or of severe high myopia during the last decade cannot be drawn, yet.

As in previous studies, the prevalence of minor high myopia and of moderate high myopia was correlated with female gender, urban region of habitation and higher school type [9–11,12,14]. Since girls as compared to boys spend more time with near work indoors and less time with outdoor activities, and since the level of education is higher in the cities than in the countryside, the association of minor high myopia and of moderate high myopia with female gender and urban region of habitation indicates the association between the prevalence of these grades of high myopia and education-related parameters.

Interestingly, the prevalence of severe high myopia (≥-10 diopters) was not significantly correlated with school type (*P* = 0.48) when adjusted for age nor was it correlated with urban region of habitation (*P* = 0.07) or female gender (*P* = 0.37), when adjusted for age. Even in univariate analysis, the prevalence of severe high myopia was not correlated with female gender, although girls as compared to boys had a significantly (*P*<0.001) higher school grade and usually perform better in school. Although the group of children with severe high myopia was relatively small (n = 87 or 0.2% of the total study population), reducing the power of a statistical analysis, the results may suggest that the prevalence of severe high myopia, as compared to the prevalence of minor high myopia or of medium high myopia, was to lower degree correlated with education-related parameters. This finding would be in contrast to the statistically strong associations of the prevalence of minor high myopia and of moderate high myopia (≥-6 diopters and ≥-8 diopters) with education-related parameters, such as higher school type (*P*<0.001 and *P*<0.001, respectively) and urban region of habitation (*P*<0.001 and *P* = 0.004, respectively). These observations would be in agreement with the result of a recent meta-analysis in which associations of high myopia in adults were compared with associations of high myopia (defined as myopic refractive error ≥-6 diopters) in school children [19]. It revealed that in the adults, education-related parameters did not show a significant association with high myopia, in particular not with high pathological myopia. In contrast, in school children, the prevalence of high myopia showed strong associations with education-related parameters [19].

Limitations of our study should be taken into account when its results are discussed. First, refractometry was not performed under cycloplegia, so that involuntary accommodation during refractometry might have covered a latent hyperopia [20,21]. Latent accommodation might also have falsely caused a low degree of myopia. In a previous study on 5,999 children with a mean age of 10.0±3.3 years (range: 4–18 years), the mean difference between cycloplegic and non-cycloplegic refractive error was 0.78 ± 0.79 diopters [21]. The difference decreased markedly with increasing myopic refractive error (*P*<0.001; standardized regression coefficient beta:0.50; regression coefficient B: 0.19; 95%CI: 0.18, 0.20) and older age (*P* = 0.006; beta: 0.04; B: 0.009; 95%CI: 0.003, 0.016). Since the focus of our study was the prevalence of high myopia in 18-year old children, it might have been unlikely that involuntary accommodation under non-cycloplegic conditions had caused a major bias in the assessment of the prevalence of high myopia in the relatively old subgroup of 18-year-old students. Second, our investigation was a cross-sectional investigation which did not allow drawing direct conclusions on a longitudinal course and causal relationship between parameters. Third, the group of children with severe high myopia was relatively small (n = 87 or 0.2% of the total study population), reducing the power of a statistical analysis. It has therefore to be taken into account, that the negative result of a lack of an association might have been due to a small statistical power. Regarding the wide confidence intervals of the relationships however may make it unlikely, that a higher number of severe highly myopic study participants would have resulted in a statistical significant association.

In conclusion, prevalence of minor, moderate and severe high myopia in 18-year-old school children in this most recent survey in a Chinese metropolitan region was 19.4%, 5.2% and 0.9%, respectively. These figures were higher than in previous surveys from mainland China. In contrast to the prevalence of minor high myopia and of moderate high myopia, the prevalence of severe high myopia (≥-10D) was not strongly correlated with educational parameters after adjusting for age. Future studies may address whether the risk of the development of pathologic myopia is bound to the severe type of high children myopia which was not strongly correlated with educational parameters.
